# Understanding the Role of Anti-PEG Antibodies in the Complement Activation by Doxil in Vitro

**DOI:** 10.3390/molecules23071700

**Published:** 2018-07-12

**Authors:** Barry W. Neun, Yechezkel Barenholz, Janos Szebeni, Marina A. Dobrovolskaia

**Affiliations:** 1 Nanotechnology Characterization Lab, Frederick National Laboratory for Cancer Research Sponsored by the National Cancer Institute, Frederick, MD 21702, USA; neunb@mail.nih.gov; 2 Membrane and Liposome Research Lab, Hebrew University Hadassah Medical School, POB 12272, Jerusalem 9112102, Israel; chezyb@gmail.com; 3 Nanomedicine Research and Education Center, Institute of Pathophysiology, Semmelweis University, 1089 Budapest, Hungary; jszebeni2@gmail.com; 4 SeroScience Ltd., Nagyvárad tér 4, 1089 Budapest, Hungary; 5 Department of Nanobiotechnology and Regenerative Medicine, Faculty of Health, Miskolc University, 3515 Miskolc, Hungary

**Keywords:** nanoparticles, liposomes, complement activation, CARPA, hypersensitivity, infusion reaction, immunotoxicity, anti-PEG antibody

## Abstract

Infusion reactions (IRs) are common immune-mediated side effects in patients treated with a variety of drug products, including, but not limited to, nanotechnology formulations. The mechanism of IRs is not fully understood. One of the best studied mechanisms of IRs to nanomedicines is the complement activation. However, it is largely unknown why some patients develop reactions to nanomedicines while others do not, and why some nanoparticles are more reactogenic than others. One of the theories is that the pre-existing anti-polyethylene glycol (PEG) antibodies initiate the complement activation and IRs in patients. In this study, we investigated this hypothesis in the case of PEGylated liposomal doxorubicin (Doxil), which, when used in a clinical setting, is known to induce IRs; referred to as complement activation-related pseudoallergy (CARPA) in sensitive individuals. We conducted the study in vitro using plasma derived from C57BL/6 mice and twenty human donor volunteers. We used mouse plasma to test a library of well-characterized mouse monoclonal antibodies with different specificity and affinity to PEG as it relates to the complement activation by Doxil. We determined the levels of pre-existing polyclonal antibodies that bind to PEG, methoxy-PEG, and PEGylated liposomes in human plasma, and we also assessed complement activation by Doxil and concentrations of complement inhibitory factors H and I in these human plasma specimens. The affinity, specificity, and other characteristics of the human polyclonal antibodies are not known at this time. Our data demonstrate that under in vitro conditions, some anti-PEG antibodies contribute to the complement activation by Doxil. Such contribution, however, needs to be considered in the context of other factors, including, but not limited to, antibody class, type, clonality, epitope specificity, affinity, and titer. In addition, our data contribute to the knowledge base used to understand and improve nanomedicine safety.

## 1. Introduction

Infusion reactions (IRs) are immune-mediated toxicities that occur within the first minutes to hours of the systemic administration of various drug products used at their relevant therapeutic doses to treat or diagnose a disease [1,2,3,4,5,6,7,8,9,10,11]. These reactions are also known as hypersensitivity, anaphylactic, or anaphylactoid reactions. In many cases, the terminology used to describe these reactions depends on the underlying mechanism [1,2,3,4,5,6,7,8,9,10,11]. However, more often than not, the exact nature of the response is unknown, and many opinions exist regarding the terminology of the reactions [3,4,5,8]. While many factors may contribute to the infusion reactions, the interactions between such factors and the cause–effect relationships among them are also commonly unknown. The infusion reactions occur in response to both traditional (e.g., low molecular weight and biologics) and complex (e.g., nanomedicines) formulations [1,8,9,12,13]. Although infusion reactions are not unique to nanotechnology-formulated drugs, they cause more concerns for nanomedicines because of the complexity of both their physicochemical attributes and the regulatory approval process. Therefore, there is an increasing need to improve the understanding of infusion reactions to nanomedicines. This need can be met by uncovering mechanisms underlying and contributing to hypersensitivity in the context of a nanoparticle’s physicochemical attributes.

Among the mechanisms underlying infusion reactions to nanomedicines is complement activation-related pseudoallergy (CARPA) syndrome [14,15,16,17,18,19,20,21]. This toxicity has overlapping manifestations and, therefore, is often confused with the immediate type I hypersensitivity reactions. Unlike type I hypersensitivity, which is triggered by drug-specific IgE, CARPA is caused by the complement activation [8,14]. The complement system is composed of approximately thirty proteins produced by the liver and present in the blood plasma to complement humoral and cell-mediated immunity against invading pathogens [22,23]. Nanomedicines may also activate these proteins and cause complement-mediated infusion reactions [8,14,18]. For example, several liposomal products (e.g., Doxil and Ambisome), micelles (e.g., Taxol and Taxotere), and iron oxides (e.g., Feraheme, Resovist, Feridex) are known to induce CARPA in sensitive patients [8,14,18,19,20]. Particle size, hydrophobicity, shape, surface curvature, the presence of cholesterol, polyethylene glycol, and doxorubicin crystals are among the factors that contribute to a nanoparticle’s ability to activate the complement system [16,20,24,25]. In addition to these intrinsic properties, some external factors (e.g., the presence of endotoxin contamination) may further contribute to the complement activation [26,27]. Moreover, several studies have described the existence of pre-existing antibodies to polyethylene glycol (PEG) [28,29,30,31,32], and their presence in one clinical study correlated with immediate-type hypersensitivity reactions to PEGylated aptamer Pegnivacogin [33]. Therefore, one of the current theories is that the pre-existing anti-PEG antibodies initiate the complement activation and IRs in patients. Because PEG is a common component in biomedical nanomaterials, there is a need to understand the potential influence of these pre-existing antibodies on both the efficacy and the safety of the PEGylated nanomedicines. Uncovering the relationship between anti-PEG antibodies and complement activation by PEGylated nanomaterials is, therefore, an essential component in the safety profile of such materials.

**Figure 1:**
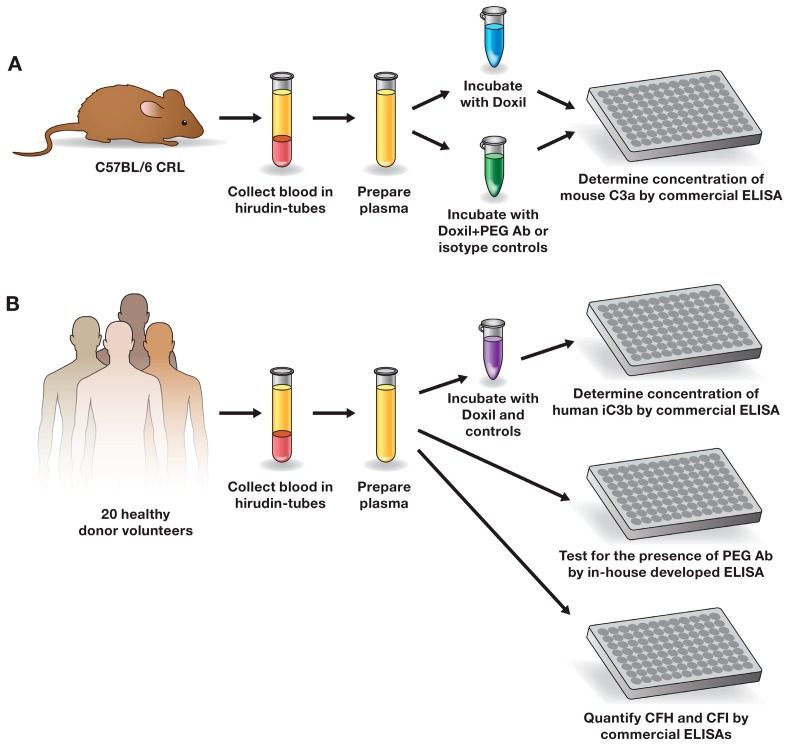
Study design. (**A**) The role of well-characterized mouse antibody in the complement activation by Doxil in mouse plasma; (**B**) detecting and understanding the role of pre-existing anti-PEG antibodies in the complement activation by Doxil in human plasma. Ab—antibody; ELISA—enzyme-linked immunosorbent assay; CFI—complement factor I; CFH—complement factor H; PEG—polyethylene glycol; CRL—Charles River Laboratories.

In this study, we used PEGylated liposomal doxorubicin formulation (Doxil) to investigate the influence of anti-PEG antibodies on the complement activation. Doxil is a nanodrug approved by the U.S. Food and Drug Administration (FDA) in 1995 and used for the treatment of over 600,000 cancer patients worldwide. Current clinical experience with this drug demonstrates that Doxil causes CARPA in sensitive patients [8,14,18,19,20]. This toxicity can also be modeled in vitro using a blood plasma assay for the assessment of complement split products, as well as in vivo using an animal model (e.g., pig) for the evaluation of the hemodynamic changes [15,26,27,34,35]. In our study, we used the in vitro assay. We tested a library of mouse monoclonal antibodies specific to various portions of PEG (backbone vs. terminal methoxy group) and representing different classes of immunoglobulins (IgG vs. IgM) to study the complement activation by Doxil in mouse plasma (Figure 1A). We also screened plasma from twenty healthy donor volunteers to identify the presence of pre-existing antibodies reactive to PEG, methoxyPEG, and PEGylated liposomal carrier of doxorubicin (herein called Doxebo); the levels of the complement inhibitory factors H (complement factor H—CFH) and I (complement factor I—CFI); and the magnitude of the complement activation by Doxil (Figure 1B). The results of our study demonstrate that anti-PEG antibodies may contribute to the complement activation by Doxil, but other factors influence the reaction and, therefore, cannot be discounted. The antibody class (IgG vs. IgM), type (pre-existing vs. derived by intentional immunization in the presence of strong adjuvants), clonality (monoclonal vs. polyclonal), titer, affinity, and epitope specificity (backbone vs. terminal group), as well as concentrations of inhibitory factors (CFH and CFI), are among such factors.

## 2. Results and Discussion

### 2.1. Mouse Monoclonal Anti-PEG Antibody May Contribute to the Complement Activation by Doxil

To explore the role of PEG-reactive antibodies in the complement activation by Doxil, we used a library of well-characterized antibodies with known class, specificity, and affinity (Table 1).

**Figure 2:**
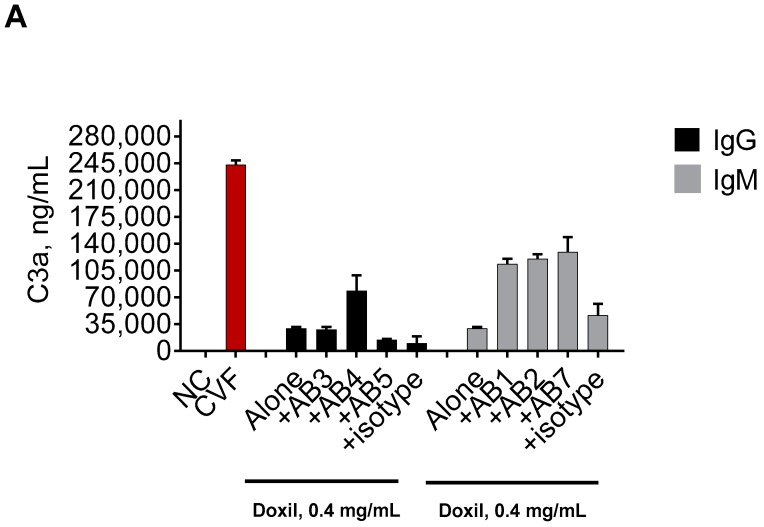

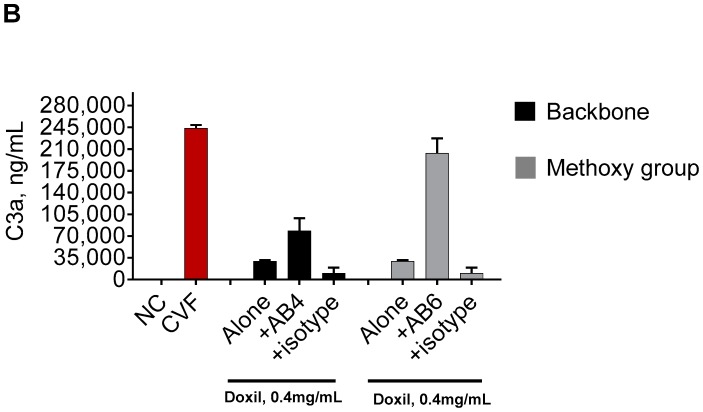
Influence of the anti-PEG antibodies on the complement activation by Doxil in murine plasma. Mouse plasma anticoagulated with hirudin was spiked with either Doxil or Doxil mixed with anti-PEG antibodies. The concentration of Doxil was 0.4 mg/mL of doxorubicin and was constant between all samples. The concentration of all antibodies was 10 μg/mL. Complement activation was assessed by ELISA measuring levels of the murine C3a component of the complement. (**A**) Role of antibody class. Doxil mixed with a PEG-specific IgM (AB1, AB2, or AB7), regardless of the antibodies’ affinity, stimulated a higher level of complement activation than Doxil alone. Doxil combined with one PEG-specific IgG (AB4) stimulated a higher level of the complement activation than Doxil alone. Doxil mixed with two other PEG-specific IgG (AB3 or AB5) induced complement activation comparable to that induced by Doxil alone; (**B**) Role of epitope specificity. Doxil incubated with IgG reactive to the PEG backbone or IgG reactive to the PEG terminal methoxy group induced stronger complement activation than Doxil alone. However, in the presence of IgG specific to the methoxy group, the complement activation by Doxil was stronger than that in the presence of PEG backbone-reactive IgG. Mouse IgG and mouse IgM, as specified in the materials and methods, were used as isotype controls and did not show a physiologically significant (i.e., two-fold or higher) influence on the complement activation by Doxil. Each bar shows mean and standard deviation (*n* = 3). All antibodies were in storage buffer containing 50% glycerol. When tested alone, 50% glycerol storage buffer did not result in C3a levels above those in the negative control (data not shown). DXR—doxorubicin; AB—antibody; numbers following “AB” refer to the antibodies described in Table 1; NC—negative control (phosphate-buffered saline—PBS); CVF—cobra venom factor used as the assay positive control.

If the binding of antibodies to the PEG component of Doxil contributed to the complement activation by this nanodrug, we expected to see higher levels of the complement split products in plasma incubated with the Doxil–PEG antibody combination than in plasma treated with Doxil alone. Phosphate-buffered saline (PBS) and cobra venom factor (CVF) were used as negative and positive controls, respectively. Potent induction of the complement system was observed with CVF (Figure 2, red bars) as compared with the PBS-treated samples (Figure 2, NC). Various anti-PEG antibodies, when added to the plasma alone, did not result in complement activation (data not shown). As the stocks of these antibodies were supplied in 50% glycerol, we also tested the storage buffer alone. Similar to the negative control and the samples with only the antibodies, no complement activation was detected in this control (data not shown). Doxil alone resulted in weak complement activation, which is evidenced by the increase in the C3a split products above that in the assay negative control (Figure 2, Alone). These data are consistent with theoretical expectations and reports in the literature [14,15,16,17,18,19,20,21,23,25,26,27,35].

We observed a more than two-fold increase in the C3a levels in plasma treated by a combination of Doxil and one out of three tested anti-PEG IgG clones (Figure 2A; compare +AB4 vs. Alone). This elevation in the C3a levels is a good indication of complement activation. No such enhancement was seen with two other anti-PEG IgG and the isotype controls (Figure 2A; compare Alone vs. +AB3, +AB4, and +isotype). Interestingly, the affinity of AB5 to the PEG backbone is stronger than that of AB4. However, only AB4 enhanced the complement activation by Doxil. It is known that the severity of the antibody-mediated toxicity depends not only on the affinity but on the functionality of the antibody [36]. It is well established that the clinical impact of the anti-drug antibodies inversely correlates with the frequency of their occurrence in that binding antibodies are more frequent than neutralizing antibodies; however, their presence in a patient’s blood causes no or less severe consequences for the patient than those of the neutralizing antibodies [37].

All three tested clones of anti-PEG IgM resulted in a more than three-fold increase in the complement activation by Doxil (Figure 2A; compare Alone vs. +AB1, +AB2, and +AB7). A slight increase in the complement activation was also observed when IgM isotype control was mixed with Doxil before the addition to plasma (Figure 2A; compare +isotype vs. Alone). It is possible that normal mouse IgM, used in this study as the isotype control, may contain pre-existing antibodies reactive to PEG, as there is an increasing number of reports demonstrating the presence of such antibodies in the blood of various animal models and humans [28,29,30,31,32]. Regardless, the magnitude of the complement activation enhancement by PEG-reactive IgM was far higher than that in the isotype control, demonstrating the positive contribution of these antibodies (Figure 2A).

The complement activation levels by Doxil in the presence of anti-PEG IgM (+AB1, +AB2, and +AB7) were also stronger (*p* < 0.05) than those in the presence of anti-PEG IgG (+AB4) (Figure 2A). These data are consistent with knowledge regarding structural differences between IgG and IgM, in that the latter antibody can bind five times more antigens and, therefore, presumably creates larger immune complexes than the IgG–Doxil complex to trigger the complement cascade [38]. Another explanation may be the difference between IgG and IgM binding to the C1q component of the complement. Specifically, it has been demonstrated that IgM has a greater capacity to bind C1q because of the different residues within C1q proteins involved in the initial recognition and final binding of this class of the immunoglobulins, as opposed to IgG [39]. Interestingly, despite the known difference in the affinity to the antigen between AB1 and AB2, no significant difference was observed in the complement activation by Doxil in the presence of these antibodies (Figure 2A, compare +AB1 vs. +AB2). These data suggest that the achieved biological effect was saturated at the tested concentration. It is possible that a difference between antibodies with different affinity may be seen if they are tested at lower concentrations; however, such experiments were not conducted in our study.

Two murine antibodies in our library belonged to the same class (IgG) and differed in specificity to the various portions of the PEG polymer. The AB4 antibody was specific to the PEG backbone, while the AB6 antibody was specific to the terminal methoxy group. AB6 resulted in stronger induction of the complement activation by Doxil than AB4 (Figure 2B). One reason for such a difference could be the better accessibility of the terminal group to the antibody. Other potential causes could be a difference in the affinity between these antibodies and a difference in the accessibility of the C1q component of the complement. It is also possible that there are other structural and functional differences between these antibodies that could contribute to the observed result.

Collectively, our data suggest that some anti-PEG antibodies can contribute to the complement activation by PEGylated liposomes. Such contribution depends on many factors, including, but not limited to, the antibody class (IgG vs. IgM), epitope specificity (terminal groups vs. backbone), and affinity. It appears that IgMs have a greater propensity to contribute to the complement activation. The anti-PEG IgG presence in plasma is insufficient to warrant complement activation by Doxil even though the antigenicity (i.e., the ability of the antibody to bind the antigen [PEG in our case]) is confirmed.

### 2.2. Plasma from Human Donors Shows Variability in the Complement Activation by Doxil and Cobra Venom Factor

**Figure 3:**
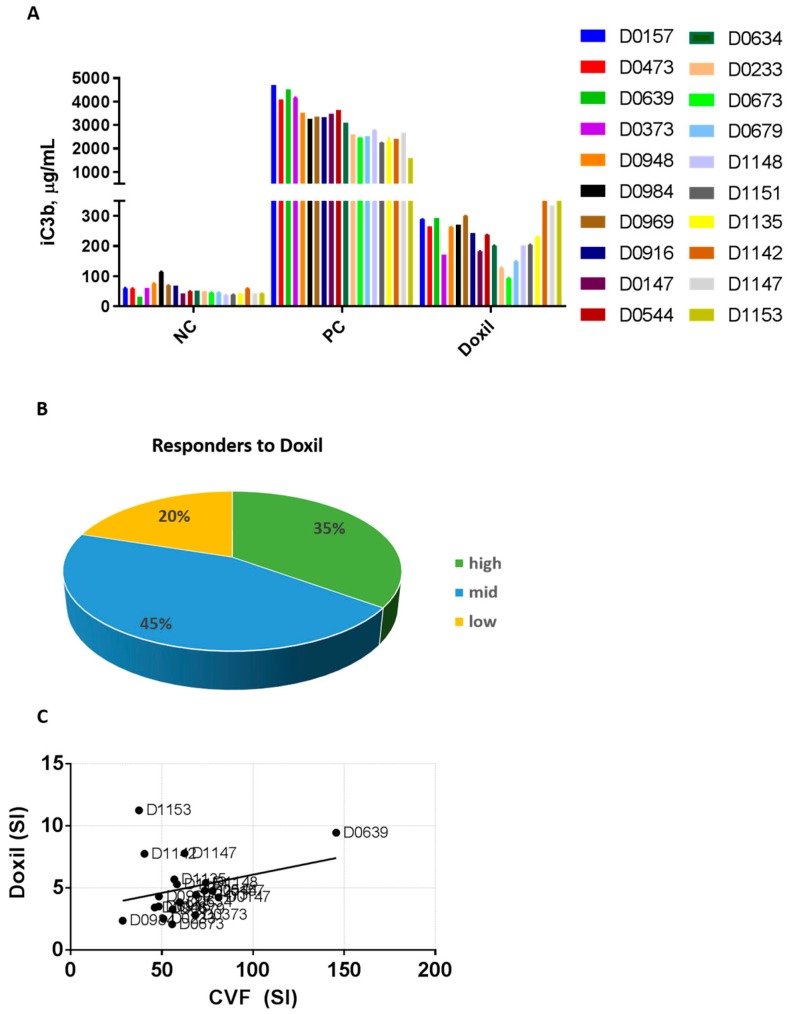
Inter-individual variability of the complement activation by Doxil in human plasma. Human plasma anticoagulated with hirudin was collected from 20 healthy donor volunteers and tested in vitro. (**A**) Plasma from individual donors was spiked with controls or Doxil and incubated for 30 min. The concentration of Doxil was 0.4 mg/mL of doxorubicin and was constant between all samples. Complement activation was assessed by ELISA measuring levels of human iC3b. Each bar shows mean response and standard deviation (*n* = 3); (**B**) Stimulation index was calculated for each donor as described in the materials and methods. Donors were grouped into three categories according to the individual magnitude of the complement activation: SI ≤ 2 was assigned as low response, SI 2–6 was assigned as medium response, and SI ≥ 6 was assigned as high response; (**C**) Regression analysis shows no linear correlation between complement activation by Doxil and CVF in individual plasma samples (*p* = 0.2112 and r = 0.08521; F = 1.677). SI—stimulation index; CVF—cobra venom factor; NC—negative control; PC—positive control (CVF); D—donor.

Not all patients undergoing therapy with PEGylated liposomal doxorubicin develop CARPA [40,41]. However, the reason for such difference in the patients’ sensitivity to Doxil is not clear. Therefore, we conducted an in vitro study using plasma from twenty human donor volunteers. We hypothesized that inter-individual variability in the complement activation by Doxil is not unique among cancer patients and is present in the plasma of healthy donors, and if so, then blood from healthy volunteers could be used for in vitro mechanistic studies. The individual plasma specimens were incubated with Doxil at the same concentration as that used in the in vitro mouse plasma study described above. All other experimental conditions, such as blood anticoagulant, incubation time, and temperature, were also kept constant. A positive response to CVF and Doxil, as judged by the at least two-fold increase in the levels of the complement split product iC3b, was observed in all tested plasma specimens (Figure 3A; compare NC vs. PC or Doxil for individual donors, who are denoted by the letter “D” and a number). However, the magnitude of the response varied between individual donors (Figure 3A). As various levels of the complement split product were also detected in the negative control, and to allow better comparison between the donors, we calculated the stimulation index (SI). The SI is the ratio between iC3b split products in the test sample and that in the negative control of the individual donor. We next performed frequency distribution analysis and grouped the donors into three categories (high, mid, and low) based on the magnitude of their response to Doxil (Figure 3B). We assigned donors with an SI higher than two but lower than six to the medium responders group, which represented 45% of our donors’ specimens. Donors with an SI of six or higher were assigned to the high responders group, which represented 35% of the donors. Donors with an SI of two or lower were placed into the low responders category, which represented 20% of the donors (Figure 3B). There is no current standard or guideline for qualifying high, mid, and low responders. The assignment into different groups presented above is based on our scientific justification, which may differ from that of other scientists.

Next, we calculated SI to the positive control (CVF) and performed regression analysis to compare it with the SI of Doxil in the same donor specimens. The strong response to CVF coincided with the strong response to Doxil in only one donor (D0639). Likewise, a weak response to both Doxil and CVF was seen in one other donor (D0984) (Figure 3C). Except for these two examples, the overall result of the regression and residual analyses did not reveal a linear correlation between the donors’ responses to Doxil and CVF (Figure 3D and data not shown). Collectively, these data suggest that factors other than polymorphism in genes encoding individual complement components may influence the complement activation by Doxil. One such factor could be the pre-existing anti-PEG antibodies.

### 2.3. Pre-Existing PEG-Reactive Antibodies are Present in Plasma of Healthy Donor Volunteers

**Figure 4:**
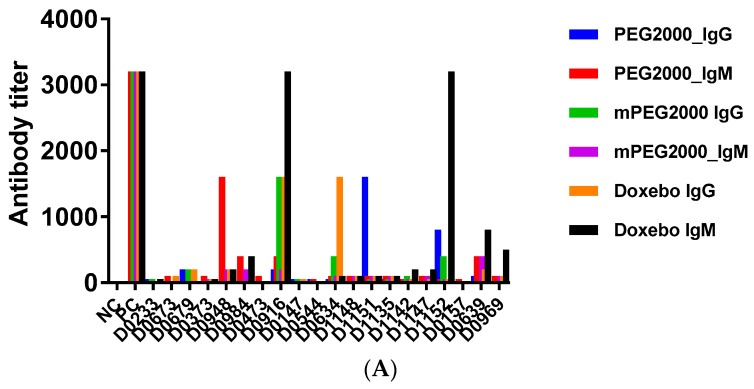

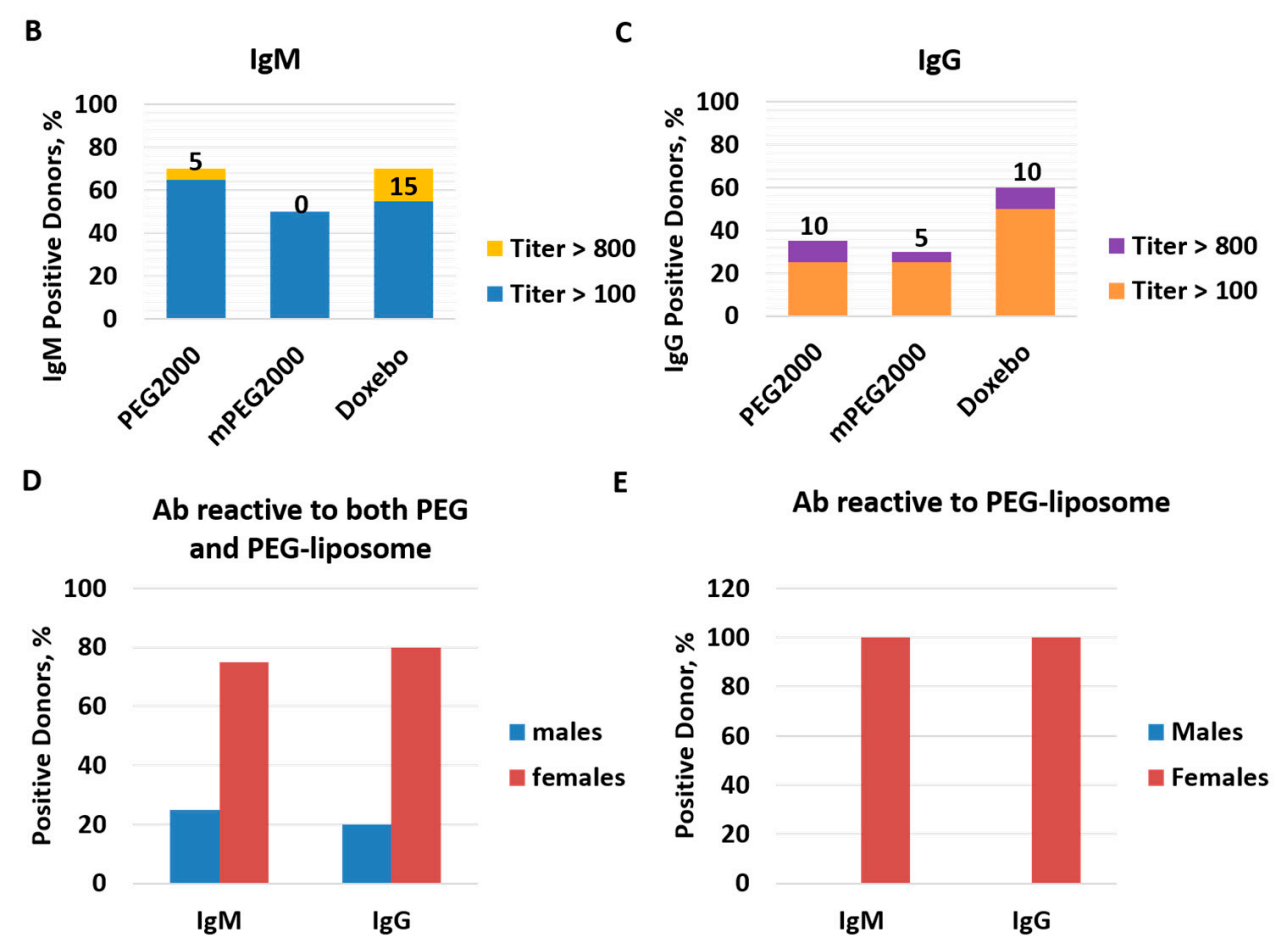
Analysis of pre-existing anti-PEG antibodies in human plasma. Human plasma anticoagulated with hirudin was collected from 20 healthy donor volunteers and tested in vitro. (**A**) A summary of antibody titers detected in individual donor plasma specimens. Positive controls are monoclonal antibodies as described in the materials and methods. There is no positive control in the PEG2000 IgG assay because currently available anti-PEG IgG are reactive to methoxy group. The titer in all shown PC samples was above 3200. Plasma from each donor was tested in duplicate on four ELISA plates. Each bar shown the titer determined from the mean response between duplicates; (**B**) Frequency of positive responders in the IgM assay; (**C**) Frequency of positive responders in the IgG assay. In both B and C, two analyses were performed. In one analysis, positive response was considered at the titer of 100 or higher. In the second analysis, the positive response was considered at the titer of 800 or higher; (**D**) Frequency of donors with antibodies reactive to both PEG and PEG-liposomes; (**E**) Frequency of donors with an antibody reactive to PEG-liposomes only. In both D and E, the positive response was considered at the titer of 800 or higher.

To investigate if the donor plasmas used in our study contain antibodies reactive with PEG, we screened all plasma specimens using in-house ELISA. The protocol for this assay has been published earlier [31]. In this assay, we use four plates to screen the individual plasmas for the presence of PEG-reactive antibodies. One plate does not contain the antigen but is processed in the same way as the antigen-coated plates. This plate is used to rule out passive adsorption of the plasma immunoglobulins to ELISA plates. Three other plates are coated with one of the following antigens: PEG, methoxy-PEG (mPEG), or PEGylated liposome (Doxebo). The molecular weight of PEG and mPEG is 2000, and it matches that used in Doxil and Doxebo. The mPEG antigen can bind antibodies reactive to both the PEG backbone and the terminal methoxy group. However, the PEG antigen can only detect the antibody reactive to the backbone. The Doxebo antigen can bind antibodies specific to the PEG backbone, the terminal methoxy group, the lipids used to make the liposome (cholesterol, phosphatidylcholine, and phosphatidylethanolamine), and a linker connecting PEG with phosphatidylethanolamine. We did not use Doxil as an antigen in this study because the presence of the doxorubicin creates the higher background and interferes with the assay readout. We describe the selection of the assay cutoff in the materials and methods. We observed that a background resulting from the nonspecific interactions between plasma proteins and assay reagents was eliminated after dilution 25. Various titers of PEG, mPEG, and Doxebo-reactive antibodies were detected in the individual samples (Figure 4A). Monoclonal antibodies spiked into pooled human plasma, which were used as the assay positive controls, demonstrated very high titers (>3200) (Figure 4A). Next, we performed two analyses that relied on different criteria of the positive response. In one analysis, we considered the antibody titer of 100 or higher as a positive response. In the second analysis, the titer of 800 or higher qualified as a positive response.

In the first analysis, plasma from more than 50% of donors contained PEG, mPEG, and Doxebo-reactive IgM (Figure 4B). In the second analysis, 5% and 15% of donors contained IgM reactive to PEG and Doxebo, respectively, while no donor specimens demonstrated a positive antibody titer to mPEG (Figure 4B). Likewise, in the first analysis, over 20% of donor specimens were positive for IgG reactive to PEG, mPEG, and Doxebo; in the second analysis, the percentages of the donor specimens with IgG reactive to PEG, mPEG, and Doxebo were 10, 5, and 10, respectively (Figure 4C). Previous studies reported that anti-PEG antibodies are more frequently seen in females [31]. In our study, the percentages of donors with PEG- and Doxebo-reactive IgG and IgM at the titer of ≥100 were higher in females than in males (Figure 4D). In the second analysis, when we applied the more stringent criterion to the positive response (i.e., the antibody titer of ≥800), females comprised 100% of the donors with IgM and IgG reactive to PEGylated liposomes (Figure 4E).

The gender-dependent prevalence of PEG-reactive antibodies observed in our study is in agreement with the earlier report [31]. The difference in the percentage of donors positive in IgM and IgG binding to PEG and mPEG suggests that most of the antibodies detected in our assay are reacting to the PEG backbone. However, we did not perform studies to confirm the epitope specificity. Therefore, purification and additional analysis of these pre-existing antibodies are needed to confirm this hypothesis. The higher percentage of plasma samples reactive to Doxebo is consistent with the expectation that this antigen may bind antibodies reactive not only with PEG. This expectation is supported by the earlier studies demonstrating the existence of naturally occurring anti-cholesterol and anti-phospholipid antibodies [42,43,44]. As Doxebo contains cholesterol, phosphatidylcholine, and phosphatidylethanolamine, it is possible that some of the donor plasmas tested in our study also contain antibodies reactive to these lipids. However, to verify this hypothesis, we would need an additional study, which was out of the scope of the current investigation.

### 2.4. Complement Activation by Doxil in Healthy Human Plasma Does Not Correlate with the Titer of Pre-Existing PEG-Reactive Antibodies

**Figure 5:**
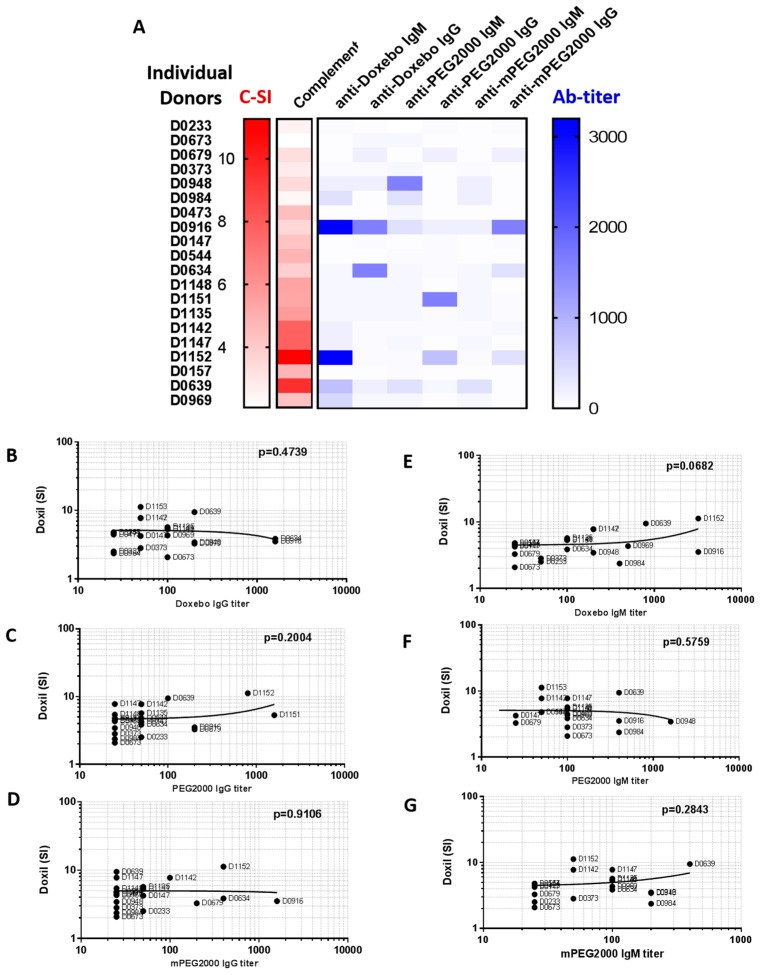
Complement activation by Doxil does not correlate with the titer of antibodies reactive to PEG in individual human donors. (**A**) The complement activation and antibody titer data are presented in a heat map format. The complement stimulation index by Doxil (C-SI) is aligned with the antibody titer (Ab-titer). Darker red and blue show higher levels of the complement activation and antibody titer, respectively. The antibody titer is plotted for IgM and IgG and indicates the antigen used to capture the reactive antibodies (Doxebo, PEG, or mPEG). The molecular weight of PEG is 2000 and is equal in all samples. mPEG contains a methoxy terminal group that is also present in the PEG2000 used to produce Doxil and Doxebo. D—donor; (**B**–**G**) Regression analysis was performed to understand the correlation between the antibody titer and the complement activation by Doxil. Doxil stimulation index (SI) was compared with the titer of IgG reactive to Doxebo (**B**), PEG2000 (**C**), or mPEG2000 (**D**), as well as the titer of IgM reactive to Doxebo (**E**), PEG2000 (**F**), or mPEG2000 (**G**). *p*-value was used to assess the significance of the confidence interval of 95%. No analyses revealed a significant correlation. The residual analysis conducted in parallel with this test did not change the conclusions of the analysis (data not shown).

To understand the functional significance of the pre-existing antibodies reactive to PEG, mPEG, and PEGylated liposomes, we compared antibody titers with the stimulation index determined in our complement activation study with Doxil. This comparison revealed one donor (D1152) who demonstrated the highest level of the complement activation by Doxil and who contained the highest titer of the Doxebo-reactive antibody (Figure 5A, D1152). However, another donor with a Doxebo-reactive IgM level as high as D1152 showed modest complement activation by Doxil (Figure 5A, D0916). Subsequent regression analysis confirmed that there is no linear correlation between PEG-reactive antibodies and the magnitude of the complement activation by Doxil (Figure 5B–G). These data suggest that the mere presence of the pre-existing polyclonal antibodies reactive with PEG, mPEG, or PEGylated liposomes may not accurately predict the complement activation by Doxil in vitro. This in vitro finding, however, cannot be directly extrapolated to the in vivo situation. Therefore, these data warrant further investigation. One way of verifying this finding is to use the plasma from patients with known in vivo IRs to Doxil and assessing the levels of anti-PEG antibodies. The difference between results obtained in mouse and human plasma can be explained by the difference of the antibodies analyzed in these experiments. Particularly, in mouse plasma we tested well characterized, high affinity, and high concentration of the monoclonal antibodies generated intentionally through careful immunization and selection procedure. In contrast, in human plasma, we study polyclonal pre-existing antibodies with unknown characteristics and a priori lower concentrations than those of the mouse monoclonal antibodies.

### 2.5. Plasma Levels of the Complement Inhibitory Factors I and H Do Not Correlate with Complement Activation

**Figure 6:**
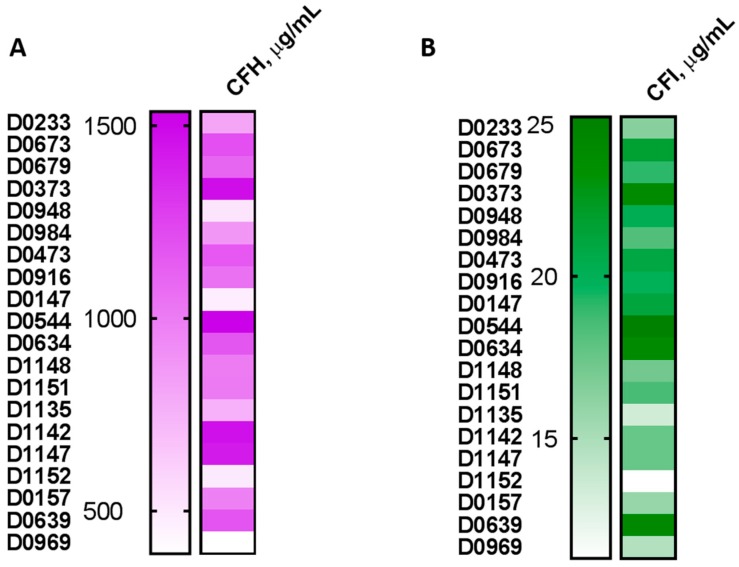

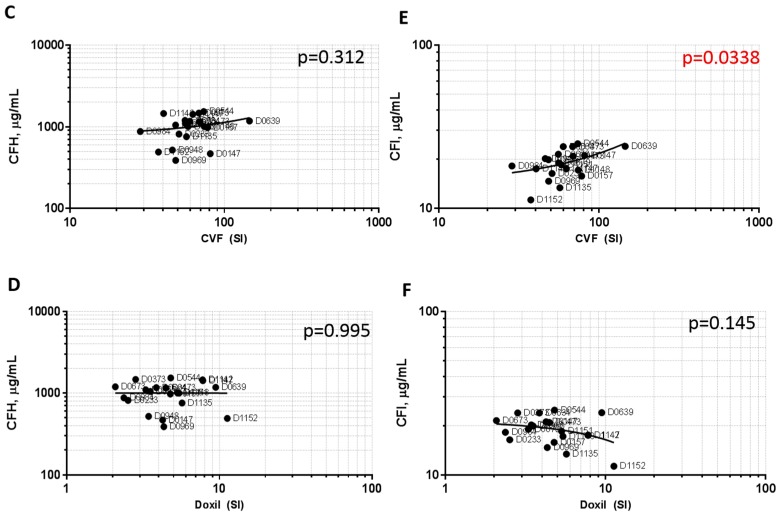
Plasma levels of complement inhibitory factors and their role in the complement activation. Plasma collected from 20 healthy donor volunteers and used in the complement activation and antibody titer study was also analyzed for the levels of CFH (**A**) and CFI (**B**). Regression analysis was performed to understand the correlation between the concentration of CFH and the complement activation by CVF (**C**) or Doxil (**D**). Regression analysis was also performed to understand the correlation between the concentration of CFI and complement activation by CVF (**E**) or Doxil (**F**). The *p*-value indicates a significant positive correlation between CFI concentration and the complement activation by CVF. No correlation was observed between CFH concentrations and complement activation by both Doxil and CVF, nor was it observed between CFI concentrations and the complement activation by Doxil.

Some proteins in the complement cascade (i.e., CFH and CFI) perform the function of the negative regulation of the complement system [45,46,47]. The higher sensitivity of monkeys than humans to the complement activation-mediated hypersensitivity reactions triggered by therapeutic oligonucleotides has recently been attributed to the differences in the levels and function of CFH between these species [48]. Genetic variability in the human CFH gene has been linked to the increased sensitivity of some people to macular degeneration and coronary heart disease [48,49,50,51,52,53]. Likewise, gene polymorphism in the human CFI gene has been implicated in hemolytic uremic syndrome and age-related macular degeneration [54,55,56,57,58]. Therefore, the available evidence suggests broad genetic diversity between individuals regarding the complement regulatory factors. The inter-individual variability in the levels and function of CFH and CFI could also influence the donor’s response to a complement-activating substance and potentially explain the inter-donor variability in the complement activation by CVF and Doxil that we observed in this study. Therefore, we next determined the concentrations of CFH and CFI in the plasma samples of the same twenty donors that were used in the complement and anti-PEG antibody experiments. Plasma levels of CFH and CFI varied between individual donors (Figure 6A,B, respectively). The concentrations of CFH did not correlate with the magnitude of the complement activation by either CVF or Doxil (Figure 6C,D). The regression analysis confirmed a positive correlation (*p* < 0.05) only between CFI concentrations and the magnitude of the complement activation by CVF (Figure 6E). The CFI levels did not correlate with the complement activation by Doxil (Figure 6F). These data demonstrate that, similar to anti-PEG antibody levels, knowing the concentrations of CFH and CFI cannot predict complement activation by Doxil in vitro. Other factors, for example variability in C1q [39], could also vary between individuals and contribute to the inter-individual variability of the response. This observation is consistent with the current notion of the multi-causality of the infusion reactions to nanomedicines [15].

## 3. Materials and Methods

### 3.1. Reagents

Hirudin blood collection tubes were purchased from Roche SSC Budapest (Budapest, Hungary). PEG2000 and mPEG2000 were from Sigma (St. Louis, MO, USA) and Laysanbio (Arab, AL, USA), respectively. Donkey anti-human IgG-HRP, rabbit anti-human IgM-HRP, goat anti-rat IgM-HRP, and donkey anti-rabbit IgG-HRP were from Jackson ImmunoResearch Labs (West Grove, PA, USA). Carnation nonfat dry milk was from Nestlé (Arlington, VA, USA). BupH tris-buffered saline and carbonate-bicarbonate buffer packs were from Pierce (Waltham, MA, USA). Anti-PEG antibodies (AGP3, AGP4, AGP6, E11, 3.3, 6.3, and 15-2b) were purchased from Academia Sinica (Taipei, Taiwan). Anti-PEG antibodies PEG-B-47 and ANPEG-1 were purchased from Epitomics/Abcam (Cambridge, MA, USA) and ANP Tech (Newark, DE, USA), respectively. Doxil and Doxebo were purchased from Avanti Polar Lipids (Alabaster, AL, USA). According to the material certificate of analysis, the concentration of the anticancer drug doxorubicin was 2 mg/mL in the Doxil formulation. Both Doxil and Doxebo had comparable total lipid concentrations. Physicochemical characterization of these particles included hydrodynamic size by dynamic light scattering, zeta potential, and endotoxin contamination by Limulus Amoebocyte Lysate assay. We previously published the results of this characterization [59]. The materials from the same lot numbers as described by us in a previous study [59] were used for this study. Therefore, we do not present these data in the present manuscript. For the complement activation assay, the tested final concentration was 0.4 mg/mL of doxorubicin. This concentration is the highest that can be achieved in vitro when Doxil is used directly from the commercial stock. It was used to mimic the conditions of the infusion reactions caused by a high local concentration at the time of injection. Human iC3b and mouse C3a ELISA kits were from Quidel (cat. #A006, San Diego, CA, USA) and Tecomedical AG (cat. #TE1038, Sissach, Switzerland), respectively. CFH and CFI ELISA kits were from HyCult Biotech (cat. #HK342-02 and HK355-02, respectively; Plymouth Meeting, PA, USA).

### 3.2. Research Blood

Human blood specimens were collected from twenty healthy donor volunteers under NCI at Frederick Protocol OH99-C-N046. Mouse blood was collected from C57BL/6 Charles River Laboratories (CRL) mice. Both human and murine blood was collected in tubes containing hirudin as an anticoagulant. Plasma specimens were prepared by blood centrifugation at 2500× *g* for 10 min, after which they were aliquoted and stored at a nominal temperature of −20 °C before use. Human plasma from individual donors was used. Mouse plasma from individual animals was pooled before use. An analysis of all experimental endpoints (i.e., complement activation, PEG-reactive antibodies, and complement inhibitory factors) was performed on the specimens from the same batch (i.e., collected on the same day and processed, stored, and handled under equivalent conditions).

### 3.3. Complement Activation

The procedure described earlier [19,27] was followed. Briefly, plasma was prepared from freshly drawn human or murine blood anti-coagulated with hirudin. Plasma was combined with test samples and controls at a volume ratio of 4:1 and incubated for 30 min at 37 °C. At the end of the incubation, all test specimens were analyzed for the presence of the complement split products (iC3b for human specimens and C3a for mouse specimens) using a commercial enzyme immunoassay kit. CVF and PBS were used as a positive and negative control, respectively. The final concentration of CVF in plasma was 10 IU/mL. For in vitro experiments involving mouse anti-PEG antibodies, the antibodies concentration was 10 μg/mL in all tested samples. The stimulation index for each donor sample was calculated by dividing the result in the test sample by the result in the negative control sample for that donor specimen. Because phlebotomy is known to induce spontaneous increase in the complement split products, to avoid false-positive background problems in the in vitro assay using human plasma, we discarded the first 10 mL of the blood drawn from human donors. Such discard is unfeasible when dealing with the mouse blood. Therefore, the assay threshold was determined in the mouse plasma samples by screening untreated plasma in the commercial C3a kit to determine the baseline. The mean optical density (OD) was determined from at least six reads, and this mean plus three standard deviations was used to set up a threshold. The results obtained with all test samples and controls were evaluated after subtraction of this threshold value so as to exclude the spontaneous complement activation that occurred in the mouse plasma sample during the specimen collection and that was unrelated to the in vitro treatments analyzed in the study.

### 3.4. Detection of PEG-Reactive Antibodies

The procedure described earlier [31] was used with slight modifications introduced to comply with current U.S. Food and Drug Administration (FDA) guidance for the immunogenicity assessment [60] and were as follows. Capture and control antibodies are summarized in Table 1. All incubations were performed at room temperature for 1 h, except for the plate coating step, which was conducted at 4 °C overnight. Nunc Maxisorp plates were coated with mouse anti-PEG IgM (AB7) and blocked with blocking buffer containing 5% nonfat milk in PBS. This antibody was used to capture antigens PEG2000, mPEG2000, or Doxebo. These antigens were omitted on the uncoated control plate. After the antigen capture, all plates (antigen-coated and uncoated) were washed once with PBS. Next, 240 µL of the dilution buffer A (2% milk in PBS) was added to all wells in row A, and 125 µL of the dilution buffer B (4% fetal bovine serum and 2% milk in PBS) was added to all other wells. Next, 10 µL of the test plasmas or positive controls was added to row A. Each sample was tested in duplicate. Serial two-fold dilutions were then performed by transferring 125 µL of the content of wells in row A to wells in row B, and so on. After one hour of incubation at room temperature, the plates were washed twice with 0.1% CHAPS in PBS and incubated with detection antibodies as follows: IgG plates received anti-human IgG-HRP, IgM plates received anti-human IgM-HRP, wells containing positive-control IgG received anti-rabbit IgG-HRP, and wells containing positive-control IgM received anti-rat IgM-HRP. All conjugates were selected so that they had minimum cross-reactivity with immunoglobulins from other classes and other hosts. The positive IgG and IgM controls were prepared by spiking 10 µg/mL of AB8 or AB9, respectively, into the pooled plasma prepared by mixing equal volumes of 20 individual plasma samples. There is no commercially available PEG backbone-specific IgG; therefore, the only assay in which the positive control was omitted was the anti-PEG IgG with PEG2000 as the antigen. After one hour of incubation at room temperature and a wash with 0.1% CHAPS in PBS, the plates were developed using TMB substrate and the optical density was determined using a plate reader set at 450 nm. The mean OD value was then calculated using readouts of the uncoated plate. This mean OD plus three standard deviations was used as the assay threshold specific to each individual donor. OD levels in the test samples of the corresponding donor on coated plates were compared with the assay threshold calculated for the same donor based on the results of the uncoated plate. The highest dilution of the test sample demonstrating the OD value above the threshold was used as the titer of antibody in that sample.

### 3.5. Determination of CFH and CFI Plasma Levels

Analyses were performed using commercial kits and according to the manufacturer’s instructions.

### 3.6. Statistical and Data Analysis

Analyses were performed using GraphPad Prism 7 software (GraphPad Software, La Jolla, CA, USA).

## 4. Summary and Conclusions

Our study demonstrated that some but not all purified, high-titer monoclonal anti-PEG antibodies contribute to the complement activation by Doxil in vitro in mouse plasma. Mouse monoclonal PEG-reactive IgM appeared as the most potent contributors. We also detected various levels of pre-existing antibodies in plasma from human donors. In our study, we used the assay that was reported previously [31]. We further optimized this assay based on the current U.S. FDA recommendations for immunogenicity assay development and validation [60]. We note that the results of our study may be different from those of other studies detecting the presence of the PEG-reactive antibodies using a different assay format, because there is currently no universal agreement on the assay format for the detection of such antibodies. Nevertheless, the findings of our study performed in the plasma of 20 donors are similar to those reported earlier in plasma from a cohort of 1400 donors [31]. That study [31] and our analysis are based on the similar assay format, which explains the agreement in the observed trends regarding the greater prevalence of the antibodies in females than in males. When we compared the levels of the pre-existing anti-PEG antibodies and the magnitude of the complement activation by Doxil in the plasma from the same donors, we did not find a direct correlation. The difference between the results obtained in mouse and human plasmas is not unexpected, because the mouse study used monoclonal antibodies generated through the specific immunization procedure, and careful selection and characterization. The mouse antibodies are monoclonal, and have higher affinity and high concentrations (10 μg/mL). The study conducted in human plasma deals with pre-existing polyclonal antibodies with unknown characteristics and specificity. Nevertheless, when these findings are considered in context with currently available knowledge regarding the complement activation by nanoparticles, several important notions can be obtained. First, it has been demonstrated that the blood from a donor who develops CARPA to Doxil in vivo also demonstrates elevated levels of the complement split products when treated with this formulation in vitro [40]. Second, it has also been reported that detectable low (approximately two-fold) levels of the complement split products are present in the plasma of Doxil-treated patients who do not experience clinical symptoms of CARPA [19], while high levels (five-fold and above) of the complement split products are detected in the plasma of patients symptomatic for CARPA [40]. CARPA is observed in vivo in approximately 5–10% of patients [40]. The occurrence of high-titer (>800) PEG-reactive IgM in our study was also 5–10%. Therefore, it is possible that, in order to observe a correlation between the pre-existing anti-PEG antibodies, we have to expand the number of donors so as to select only those whose plasma contains high titers of the anti-PEG IgM, and then repeat the analysis. Because there is no current standard regarding how high the titer of the anti-PEG antibody should be in order to increase the in vivo risk of CARPA, and because the existing in vitro data regarding antibody titers are not yet supported by the clinical results in CARPA patients, we suggest that conducting a functional assay in which a patient’s plasma is treated with Doxil in vitro and assessed for the presence of complement split products may be more accurate for identifying patients prone to CARPA than screening their blood for the presence of pre-existing anti-PEG antibodies. This recommendation is supported by the existing knowledge base that includes both in vitro data on the complement split products, as well as clinical and in vivo animal model results of CARPA and plasma levels of the complement split products. Obviously, more research is needed to establish the levels of the PEG-reactive antibodies that serve as prerequisites of CARPA. Other factors, such as antibody class, affinity, and epitope specificity, as well as concentrations of plasma CFH and CFI levels, also need to be considered and are in line with the current multi-causal nature of the nanomedicine-mediated hypersensitivity reactions [15]. Moreover, a recent study demonstrated that the binding of the complement proteins to one nanomedicine could not predict the complement binding to another nanomedicine, even when the study is conducted using blood from the same individual [61]. Therefore, if a functional assay is used to identify patients prone to CARPA, such testing should be repeated for each nanomedicine in question, as the data obtained with one nanomedicine cannot be directly extrapolated to predict a patient’s response to another nanomedicine. Collectively, the available evidence suggests that such functional tests need to be conducted for each patient and each type of the nanomedicine. Finally, the results of our in vitro study suggest that a verification with clinical data is necessary. To verify the relevance of this in vitro study to the clinical scenario, one has to conduct a clinical trial with cancer patients treated with Doxil. In such a clinical trial, the type and level of anti-PEG antibodies, level of complement activation biomarkers, and IRs would need to be studied concomitantly with the in vitro assays similar to those described in our study.

## Figures and Tables

**Table 1:** Library of anti-polyethylene glycol (PEG) antibodies. All antibodies were obtained from commercial suppliers. The attributes listed in the table are summarized based on the certificate of analysis supplied with the antibodies. Antibodies 1–6 were used in the in vitro complement activation study in mouse plasma. Antibodies 7–9 were used in the in vitro assay applied for the detection of pre-existing anti-PEG antibodies in human plasma. According to the manufacturer, the affinity of antibodies varied as follows: AB1 < AB2 and AB3 < AB4 < AB5. Relevant isotype controls that were used in this study are provided in the materials and methods. AB—antibody; -OCH3—terminal methoxy group.

Number	Antibody	Host	Type	Epitope Specificity
AB1	AGP3	Mouse	IgM	Backbone
AB2	AGP4	Mouse	IgM	Backbone
AB3	E11	Mouse	IgG1	Backbone
AB4	3.3	Mouse	IgG	Backbone
AB5	6.3	Mouse	IgG1	Backbone
AB6	15-2b	Mouse	IgG1	-OCH3
AB7	ANPEG-1	Mouse	IgM	Backbone
AB8	PEG-B-47	Rabbit	IgG	-OCH3
AB9	AGP6	Rat	IgM	Backbone

## Terminology

The term “pre-existing” was used in this manuscript to describe anti-PEG antibodies detected in plasma of human donor volunteers without known prior exposure to PEGylated liposomes. Other known terminologies used to describe these antibodies are “naturally occurring”, “natural antibodies of environmental origin” and “environmentally-induced”.
